# Precise Engineering of Sustainable Fiber Composites Comprising Biobased or Valorized Matrices and Solid Components: A Perspective

**DOI:** 10.1021/acsenvironau.6c00056

**Published:** 2026-07-13

**Authors:** Vojtěch Jašek, Radek Přikryl

**Affiliations:** Institute of Materials Chemistry, Faculty of Chemistry, 48274 Brno University of Technology, 61200 Brno, Czech Republic

**Keywords:** composites, sustainability, recycling, biobased compounds, valorizing, fibers

## Abstract

Long-fiber composites play a significant role in a wide range of applications across industrial sectors, including automotive, construction, energy, aerospace, and marine. Current trends point to sustainable solutions that use environmentally friendly materials, reduce waste, rely on renewable energy sources, and adopt circular manufacturing. Reusing, valorizing, and upcycling ensure an optimal approach to economically rational solutions connected to environmentally friendly procedures. Long-fiber composite materials contain multiple functional components beyond solid-phase filament reinforcement, including a continuous binding resin (matrix), solid-phase particle fillers, separators, and functional additives. From a sustainability standpoint, the long-fiber reinforcements and the binding matrix are key components, as they account for the majority of a composite’s total weight. This work summarizes, describes, and evaluates several approaches to valorizing, circulating, and reusing either the fiber or the matrix composite content. The sustainability aspect can be enhanced by circulating composite products, modifying and reusing functionalized resins, incorporating natural fibers as alternatives to fossil-based ones, or engineering novel, partly or fully biobased matrices as substitutes for currently used petroleum resins. The primary aim of this work is to provide a comprehensive and critical evaluation of the numerous strategies reported in the available literature and to determine the most promising sustainable strategies.

## Graphical abstract

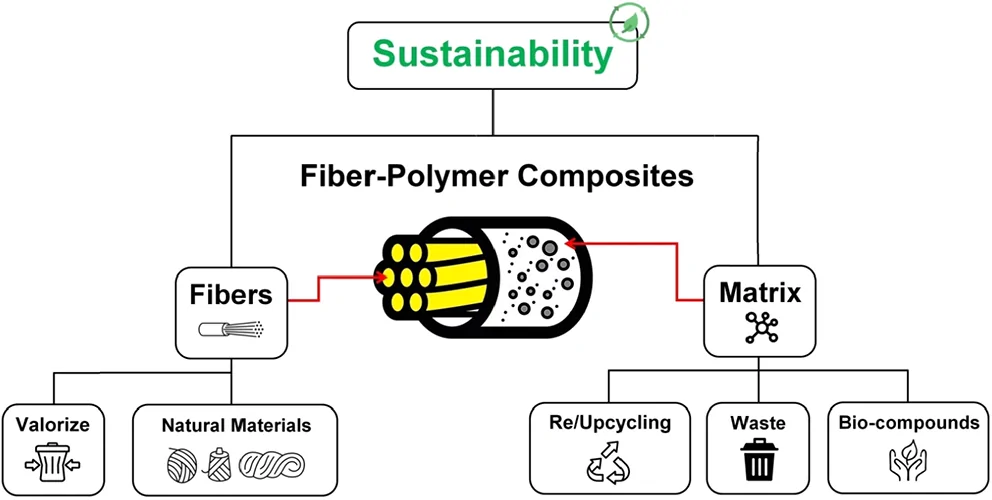

## 1 Introduction

Sustainable approaches are increasingly required across various material fields and applications in industry, driven by new legislation and green policies.^1,2^ Composites and heterogeneous materials are much more complicated materials due to the multiple factors. Laminates and multimaterial systems contain a wide spectrum of solid fillings, reinforcements, and the binding matrix (see Figure 1).^3–5^ The continuous fibers account for a major weight fraction in the formed composites.^6^ The most applied reinforcement types are glass fibers (typically categorized as E-glass (standard)^7^ and S-glass (high strength)),^8^ carbon fibers,^9^ and aramid (polyamide—Kevlar).^10^ The most used fillers in commercially produced composites are mineral-origin (calcium carbonate or silica) due to their high availability, low cost, and functional importance (carbonates represent bases which can neutralize the potential acidic matrices applied in the composite manufacture).^11–13^ Moreover, these natural fillers reduce the composite’s shrinkage and improve eventual thermal stability.^14^ Other, more expensive fillers are used in the industry, such as metallic (conductivity enhancement),^15^ ceramic particles (hardness boost),^16^ and microspheres (weight reduction).^17^ Several specific and functional additives are applied in specific composites to provide particular properties, like toughness increase (rubber particles),^18^ interlaminar shear strength boost (carbon nanotubes),^19^ and flame retardant character (antimony trioxide or alumina trihydrate).^20,21^ Regarding sustainability, the reinforcement options are the most promising solutions, offering improvements in the properties of renewable composites. Several studies have explored various approaches to incorporating natural reinforcement fibers and fabrics, including flax, bamboo, hemp, jute, pineapple leaf, and cotton.^22–27^ The selective recycling/upcycling of the residual or produced particles and the manufacturing scrap may also increase the sustainability factor in production, since the nonrenewable solid reinforcements may remain in composites. However, their waste products can be incorporated into the final products (as a powder, fraction, or blend).^28,29^ The primary challenge in enhancing the solid parts and reinforcements in composites is ensuring their final properties and quality. This perspective will provide the necessary information on the potential of renewable and natural fibers, or of valorized and upcycled solid-phase scrap, to match the benchmark properties of the composite achieved with virgin components.

**Figure 1:**
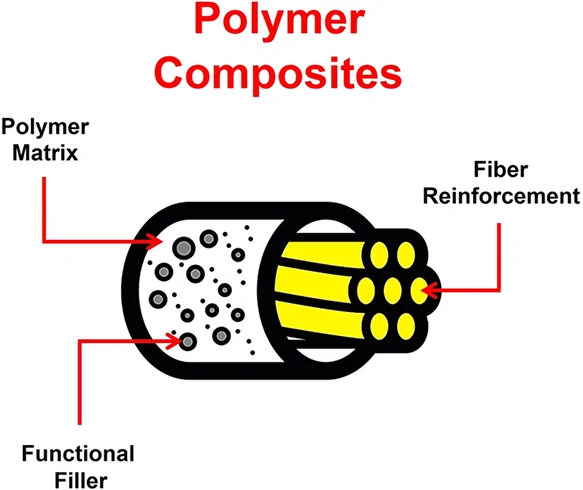
Schematic main structural components of the polymer-based composites.

Alongside several renewable, valorized, recycled, upcycled, or biobased reinforcements and solid-phase particles forming various composites, the continuous and homogeneous phases (thermoplastic or thermoset) might also be obtained from waste sources or derived from renewable compounds.^30,31^ Both strategies, circularity and the incorporation of biosources, help increase the sustainable aspect in the composite industry. Regarding thermoplastic matrices, several polymers can constitute the homogeneous composite phase, such as polyamides (PA),^32^ polypropylene (PP),^33^ polycarbonate (PC),^34^ polyethylene terephthalate (PET),^35^ and polybutylene terephthalate (PBT).^36^ Since thermoplastics are conveniently processed by mechanical upcycling (reprocessing the polymeric melt),^37^ the circularity-enhancing approach comprises the incorporation of particular scraps from primary plastics manufacture, such as modified PET, rPET, or polypropylene.^38–40^ The composite multicomponent structure allows lower requirements for the used thermoplastic matrices due to the synergistic effect.^41^ Apart from the fossil-based, recycled, and valorized polymers, composites may consist of pure biobased systems, namely, polylactic acid (PLA),^42^ polyhydroxyalkanoates (PHAs),^43^ or biobased compounds like biopolyethylene (bio-PE)^44^ or biopolypropylene (bio-PP).^45^ The thermally less stable thermoplastic polymers (biopolyesters such as PLA or PHAs) are not suitable for the mechanical recycling strategy^46,47^ due to their rapid quality decrease at higher temperatures and certain processing conditions (PHAs’ thermal degradation starts at around 180–190 °C, PLA suffers considerable hydrolytic instability).^48,49^ Instead of melting and incorporating a liquid biopolyester, suitable chemical upcycling and structural functionalization improve the specific polymers’ characteristics, thereby ensuring several benefits in the produced composites.^50,51^ PLA and PHAs comprise carboxyl and hydroxyl groups within their structure; therefore, the chemically engineered derivatives (amides, cross-linked esters, and vitrimer structures)^50,51^ may enhance several important composite properties (impact strength, interlaminar shear strength, total hydrophobicity, or improved interphase compatibility).^52–55^

While thermoplastic matrices can be reprocessed and used numerous times, thermoset polymers are also commonly used to produce laminates, despite their high stability and permanent solid form resulting from a three-dimensional macromolecular cross-linked structure.^56^ Several thermoset precursors are applied in the composite industry, like unsaturated polyesters (UPs),^57^ epoxy resins (EP),^58^ vinyl ester systems (VE),^59^ phenolic resins (PF),^60^ and reactive polyurethanes (PUR).^61^ From the thermoset circularity standpoint, the polymerization strategy is critical for the potential circularity or reprocessing of the formed composites. The radically polymerized matrices (UPs, VE) and the polycondensed thermally stable polymers (PF) represent exceptionally stable polymers composed of the covalently bonded hydrocarbon backbones.^62,63^ On the other hand, the specifically engineered epoxy resins and polyurethanes may be circularly reused if particular chemical strategies are applied. EPs can be polymerized via ionic polymerization (unsuitable for potential circularity due to the highly stable polyether structure),^64^ or with specific hardeners (amines and anhydrides are the most common), which act as reactive nucleophiles that attack the epoxy functional groups, forming amine/imine or ester bonds.^65,66^ Such polymerized precursors are much less chemically stable; therefore, their chemical backbones can be released (at elevated temperatures and pressures) to become partially reprocessable. Eventually, the released chemical structures, which contain specific reactive functional groups (hydroxyl, carboxyl, amine, epoxy), form a secondary, cross-linked three-dimensional (3D) molecular structure. Such structures, which feature dynamic covalent bonds, are called vitrimers.^67–69^ Except for the vitrimer dynamic structures, several works and investigations have introduced chemical recycling of polyurethanes via various chemical strategies—glycolysis, aminolysis, acidolysis, or hydrolysis.^70–72^ The urethane (carbamate) bond (>N–C­(=O)–O−), formed from isocyanate and alcohol, can undergo bond breakage under specific conditions (suitable catalysts or microwave-assisted decomposition),^73–75^ resulting in the substitution of the original alcohol/polyol for the chosen nucleophile (based on the chosen reactive solvent).^76^ Not only can the original polyol be isolated and recycled or valorized in any different application, but the obtained PUR raw depolymerized solution can be circularly incorporated into the virgin polyurethane system or modified via a different chemical strategy.^77^ Numerous suggested and realized processes were reported in the available literature.^70–75^ The biobased thermoset precursors can also represent more sustainable and environmentally friendly alternatives to the currently used fossil-based polymerizable systems. The reported studies uncover specific syntheses that produce biobased epoxides with the potential to replace nonrenewable systems derived from bisphenol A or glycidyl ethers.^78,79^ Numerous structures synthesized from renewable starting materials modified with acrylic/methacrylic compounds were introduced and described for composite fabrication. Such systems are polymerized via radical polymerization; therefore, their sustainability depends on the natural starting materials used.^80,81^ Itaconic acid-based systems exemplify a promising and unique group of precursors due to their curable structure and the ability to be produced entirely biotechnologically from natural sources.^82^ Itaconic acid has been widely described as a biobased alternative to acrylates and methacrylates, produced via microbial cultivation.^83–85^ The specifically engineered itaconate systems represent radically polymerizable precursors that are fully produced from renewable sources.^86^ This work will investigate which approach would work better—the reprocessed fossil-based wastes and scrap or a novel system produced via sustainable strategies.

This perspective provides a comprehensive literature review of various approaches to more sustainable multimaterial composites, including the use of valorized wastes, innovative and natural systems using renewable materials, and chemical approaches to recycling and upcycling the original molecular substances into improved composite matrices (graphically illustrated in Figure 2). The main scope of this perspective is long-fiber-polymer composites and various sustainable strategies to incorporate renewable or waste-valorized inputs. This work comprises two main subsections: solid-phase reinforcement and polymerizable matrices. The main concerns and questions are

- How complex and complicated are the alternative processes and the suggested syntheses?
- Can sustainable approaches be incorporated into industrial-scale composite production?
- Are the properties achieved by sustainable composites competitive with those of standard systems?
- What are the most suitable applications of the new composites?

**Figure 2:**
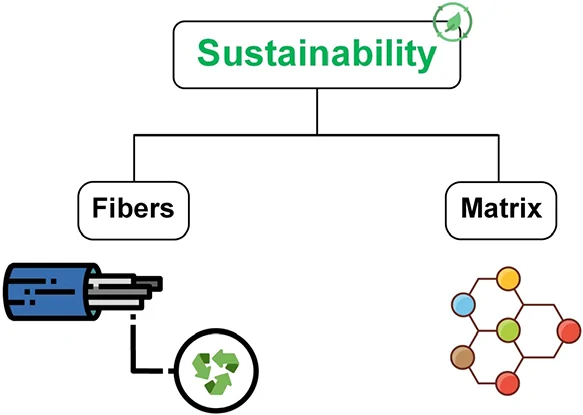
Two main components of long-fiber composites are discussed in this work from a sustainability perspective.

We will seek answers to all of these questions throughout this perspective, supported by up-to-date published investigations and experimental observations.

## 2 Sustainable Fiber Approaches

The fiber filling typically accounts for the majority of the composite weight (and volume) due to better final material properties and a lower final cost (the binding matrix is a more expensive component in glass-fiber composites, e.g., compared to the filling).^90,91^ When carbon fiber composites are considered, the filler is the most expensive component; therefore, to facilitate the recycling and valorization of waste or to add additional carbon filler, it is crucial to reduce process costs and reincorporate this valuable component.^92^ This section will evaluate potential circular and valorization approaches for reusing waste fiber fillings in new products to enhance both the sustainability of the process and the economic rationality. In addition to valorizing previously processed fibers and fabrics, using fully natural continuous fillers is an alternative to enhancing the sustainability of the composite industry. The most widely studied natural fibers, such as jute, hemp, kenaf, flax, and sisal, have the potential to produce composites and laminates with a high natural content (when a fossil-based binder is used).^121^ The studied biosourced fibers and fillings are also summarized in the following section.

Glass fiber composites dominate the composite materials sector mainly due to the high availability of solid-phase fillers, their low cost, good performance, and straightforward processing.^93,94^ As mentioned earlier, the market price of the commonly used matrices (unsaturated polyesters, vinyl esters, epoxy, polyurethane) surpasses the market expenses for the glass fiber filling.^91,95^ Therefore, the recycling of the solid-phase filling would not primarily save high expenses in the wasted glass content, but the suggested and performed valorization still boosts the circularity and the sustainability aspect of the manufacture. Also, the produced scrap may not exactly save a considerable amount of money by its valorization, but nowadays, the companies have to pay for the controlled disposal of the generated waste; that is why the incorporation of their own scrap or any waste obtained from other partners or companies may reduce the final costs indirectly by avoiding paying for the controlled disposal.^96^ Taking into account the listed arguments, several investigations proposing the incorporation of glass fiber waste were performed and published. Numerous studies available focus on the primary source of glass fiber-based composites used in large quantities—materials for wind turbine blades (see Figure 3).^97–99^ Such products contain a considerable glass content (typically 50–70 wt %), which is usually recovered by pyrolysis of the binding matrix, releasing the glass for potential valorization.^87,100^ ext to the glass separation via pyrolysis, grinding followed by a suitable purification step (e.g., a fluidized-bed recycler) represents an alternative for energy recovery from the matrix.^88,101^ Naturally, the mechanical recycling of glass scrap exemplifies a prospective approach to creating new glass fibers or fabrics for new applications. This strategy was preferred in the majority of the circular solutions described.^87,98,99^ Another alternative is to transform glass scrap or the entire composite scrap into powder form and use this material to produce new products, substituting for commonly used fillers such as carbonates, silica, or virgin glass particles.^102,103^ From a simplicity standpoint, incorporating the powder glass/composite content is associated with lower additional costs (when grinding is mostly avoided). On the other hand, the mechanical recycling of the glass waste provides a new virgin solid-phase filler, but is circulated at considerably higher processing costs (the glass melting temperature is around 1500 °C, which represents considerable expenses in the energy consumption).^104^

**Figure 3:**
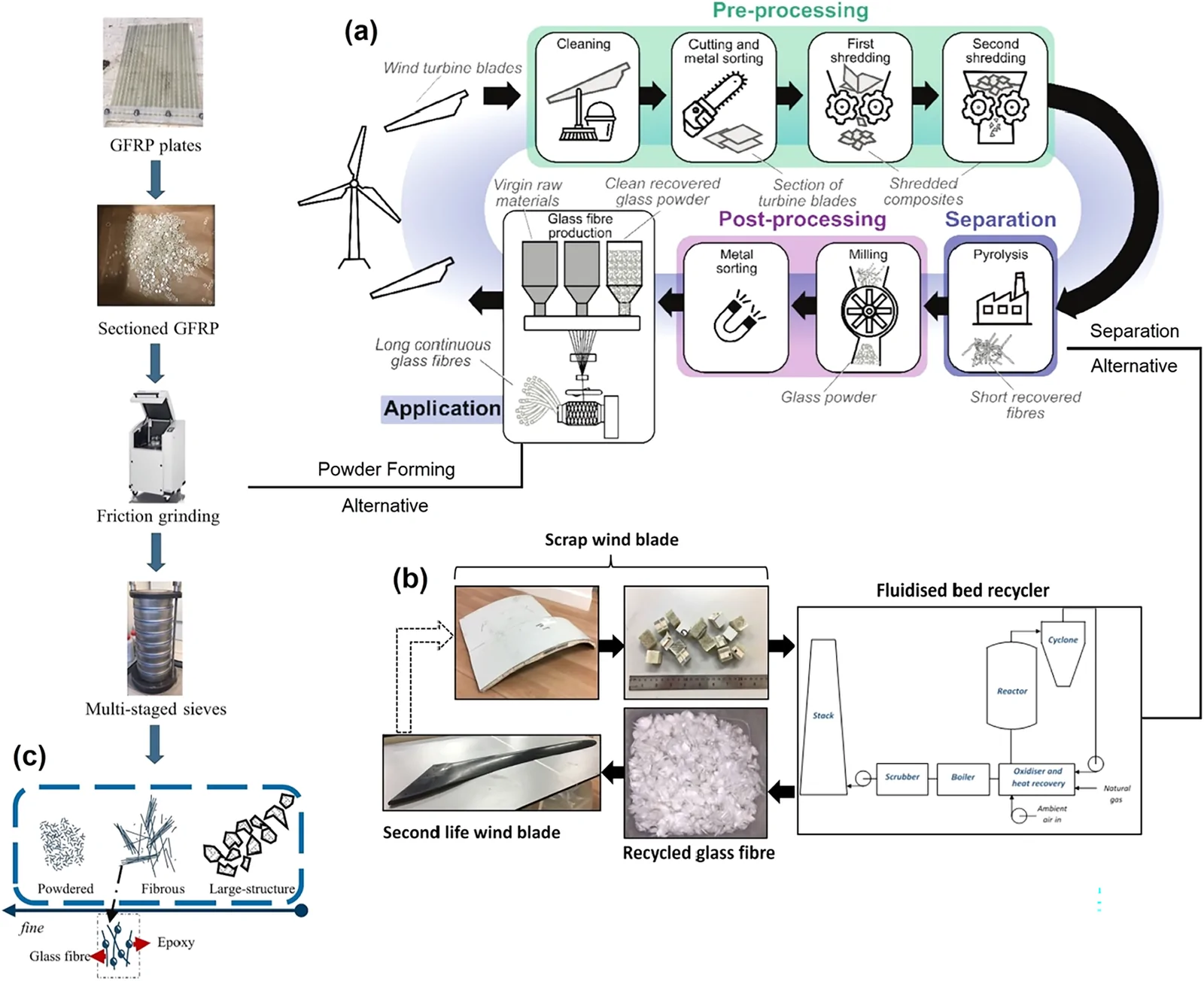
Possibilities of valorizing scrap and waste from glass fiber-containing products and composites. (a) The multistep strategy produces pure glass particles via pyrolysis separation and metal sorting, enabling mechanical recycling into glass fibers. Reproduced with permission under a Creative Commons CC-BY license from [87], Copyright 2025, Wiley. (b) The alternative purification of glass-containing preprocessed scrap using fluidized-bed recycling. Reproduced with permission under a Creative Commons CC-BY license from [88], Copyright 2024, Springer.^88^ (c) Another alternative to valorize the produced glass or the whole composite scrap into a solid-phase powder filler applicable in the composite fabrication. Reproduced with permission, Copyright 2021, Elsevier.^89^

The carbon fibers in composite materials pose a much more complex challenge for sustainability approaches due to their high cost in final products.^105,106^ Compared to the glass fibers and fabrics, the complete recycling of carbon filaments is much more complicated. Carbon polymer structures are less thermally stable than glass materials, especially in oxidizing environments. Therefore, their removal and purification of excess resin need to be more precise and engineered (rather than simple ultrahigh-temperature pyrolysis, as with glass fibers).^107,108^ Several strategies are used to purify the obtained carbon fibers or scrap, including temperature-regulated pyrolysis at 500–600 °C, fluidized-bed purification, and chemical solvolysis using supercritical fluids or low-temperature functional reactive solvents.^109–113^ The purified, ground, and mechanically recycled carbon fibers (typically in short-fiber or ground powder form) can be used as solid-phase heterogeneous fillers in new thermoset composites. Producing a fine powder comprising valorized carbon fibers exemplifies a high-performing functional reinforcing particle filler that may outperform natural powder fillers, such as carbonates, silica, or talc.^114,115^ The medium-length purified carbon fiber may form longer “fiber type” structures for valorization in several molded products; however, the properties of the circulated carbon fiber structures will always be worse than those of virgin fibers.^116^ The most optimal application fields for the recycled or valorized carbon fibers are automotive parts, sporting equipment, nonfunctional aerospace gadgets, or various reinforcements in polymer- or inorganic-based composites.^117–120^

The summarized glass and carbon high-performance fibers represent irreplaceable solid-phase reinforcements for the composite industry. The future of the materials industry could benefit from their high chemical stability and potential circularity in recovered form as the use of virgin materials decreases, transforming the manufacturing process into a sustainable one that enables the exchange of processed materials and components across different applications. In particular, when wind-blade processing is considered, the recovered solid-phase particles may be used to produce building composites, noncritical automotive parts, or design objects. Seeking circularity-enhancing approaches can build bridges among different material industries and reduce overall waste.

Natural fibers exemplify a direct alternative to virgin fossil-based or expensive solid-phase continuous fillers. Their production and manufacturing are fully based on renewable sources, as the substances used are derived from vegetable materials.^128^ In several cases, the biobased fabrics and filaments overcome the used commercial petroleum systems from the price and sustainability standpoint; however, their functional properties cannot match those of the high-performing materials, such as carbon fibers, aramid, polyamides, and others.^129^ The glass fibers, especially S-glass but also the less expensive E-glass, are commonly connected to higher overall expenses compared with the natural fibers in terms of the raw material cost. Unlike commercial polymer filaments, the functional performance of renewable natural fibers is an essential disadvantage.^130,131^ Moreover, regarding potential circularity, which was discussed in detail earlier in this Perspective, the glass and carbon fibers are, to varying degrees, capable of being recycled or valorized. On the other hand, the virgin natural fibers are practically nonrecyclable (except for grinding and filling in powder form) due to their complex chemical structure (lignocellulose organic structure that cannot be efficiently reprocessed via melting, like glass fibers, or mechanically recycled and reused with sufficient remaining properties, like carbon fibers).^132^ Taking into account all of the mentioned and summarized characteristics, the composite manufactured from natural fibers can still find its valuable application fields, where they represent a sustainable-increasing factor, together with the potential cost reduction.^131^ The lightweight character of such composites is valuable for specific car interior parts in the automotive, construction, and building sectors, which may benefit from the lower manufacturing cost of applying the natural fiber composites in interior and particular exterior components, and the sustainability-conscious customers can value the incorporation of such materials in sports equipment or biobased packaging.^133^ Various types of the most investigated natural fibers applied in material applications are summarized in Table 1.

**Table 1:** Natural Fiber Composites in Various Material Applications

type of fiber	content in composite	application	tensile strength (MPa)	substitute for	reference
jute	30%, 50–70%	automotive thermal insulation	not measured	aerogel composite, glass fiber composite	^122^
hemp	38.3%	marine applications	31.3–45.7	glass fiber composite	^123^
kenaf	75–85%	biomedical applications	94–340	biodegradable composites, ceramic composites	^125^
flax	29–51%	electric personal watercraft	34.6–42.3	carbon fiber	^124^
sisal	10–30%	automotive brake pad	not measured	ceramic composite, semimetallic composite	^126^
bamboo	12%, 24%	automotive spoiler products	30.8–35.1	carbon fiber, glass fiber	^127^

As expected, several material applications showed decreasing trends in mechanical properties, especially in tensile and flexural properties at break. The jute-bamboo-glass composites containing 57% natural fiber achieved 89–94% of the final mechanical properties compared to materials with 37–39% natural fiber content (in which the natural fiber was replaced with glass fiber). The impact strengths of the higher green composites ranged from 55% to 85%, indicating that materials with lower natural fiber content also exhibit higher impact strengths. Since mechanical performance is expected to decrease, natural-based composites should be used in suitable applications (e.g., lightweight components or decorative purposes).^134^ Another investigation, comparing natural fiber composites (flax fibers, 45–49%) with different matrices (petrochemical epoxy resin, biobased recyclable epoxy, and radically polymerized acrylic thermoplastic resin) compared to highly filled glass fiber-based materials (containing 58–60% of filling). The weathering conditions were also taken into account, with the natural-based composites retaining about 70% of the original flexural strength and 60–75% of the flexural modulus after 56 days (compared to glass fiber composites, which retained 98% of the original properties). The significantly worse properties (especially in rigid products) are the result of natural fibers’ generally lower tensile strength and poorer compatibility with the binding matrices.^135^

Since the vast majority of natural fibers are highly hydrophilic (due to their cellulose and hemicellulose content, which possess an exceptional number of free hydroxyl groups), the interphase compatibility with most commercial resins is negatively affected (common unsaturated polyesters, vinyl esters, and others are strongly hydrophobic).^136^ Moreover, as the weathering results discussed illustrate, natural fiber-based composites exhibit significant performance declines, primarily due to their water absorption and retention characteristics.^134,135^ The lightweight nature of natural fiber composites is among the most significant positive characteristics of such materials. When combined with specific biobased matrices (such as PLA), the final product may serve specific applications while retaining its renewable character and potential for degradation.^137^ The surface treatment employs several chemical strategies comprising multiple reaction steps to modify the target fiber’s surface. The silane coupling mechanism is one of the most widely used approaches for altering the structural behavior of solid-phase reinforcement in composites. First, the silane alkoxides undergo hydrolysis to form silanols. These structures with free hydroxyl groups condense to form oligomers. Such compounds adsorb onto the treated surface via intermolecular noncovalent interactions (e.g., hydrogen bonding and Keesom interactions). Finally, after the drying/curing step, the noncovalently adsorbed oligo-siloxanes dehydrate and form covalent bonds with the treated surface.^199^ Acetylation is a straightforward chemical reaction, modifying a free hydroxyl group to less-polar acetyl compounds that represent potential hydrogen-bond acceptors but lack hydrogen-bond donor character. Therefore, the acetylated solid-phase fiber exhibits lower surface energy and greater compatibility with nonpolar curable matrices.^200^

The previously mentioned water absorption, which negatively affects the functional character of the composites, may be mitigated through several chemical functionalization methods, such as alkali treatment, silanization, acetylation, or coating with hydrophobic substances (e.g., stearic acid) (see Figure 4).^138^ The treated natural fibers combine the renewable character while their disadvantages are decreased. Not only does the modification help prevent humidity absorption, but the incorporation of hydrophobic structures into the natural fiber carbon backbone also enhances compatibility with the binder resin.^139^ Interlaminar (or interfacial) shear strength (IFSS) provides information on the compatibility between the solid-phase reinforcement and the polymer matrix. IFSS is measured by the flex mechanical deformation over a short distance to investigate the separation of fiber filling from the polymerized matrix. Silanization is one of the most common surface treatments, improving the compatibility of polar fiber surfaces with commonly nonpolar matrices. The nominal ILSS values of the investigated flax fibers in epoxy resin-forming biobased composite ranged from 23.5 to 26.3 MPa,^192^ while different volume fillings of the silanized E-glass achieved ILSS values of 17.5 to 30.8 MPa.^193^ The effective difference between the nonsilanized and the surface-treated flax fibers reached 7–12% ILSS increase with the surface modification.^192^ Alkalization is another surface treatment that chemically modifies the fiber material. In the recently published study, the authors achieved ILSS improvements for jute in combination with ramie from 12.01 *±* 5.5 MPa to 18.5 ± 9.5 MPa, and for jute in combination with sisal from 13.15 *±* 4.4 MPa to 26.56 ± 5.1 MPa.^194^

**Figure 4:**
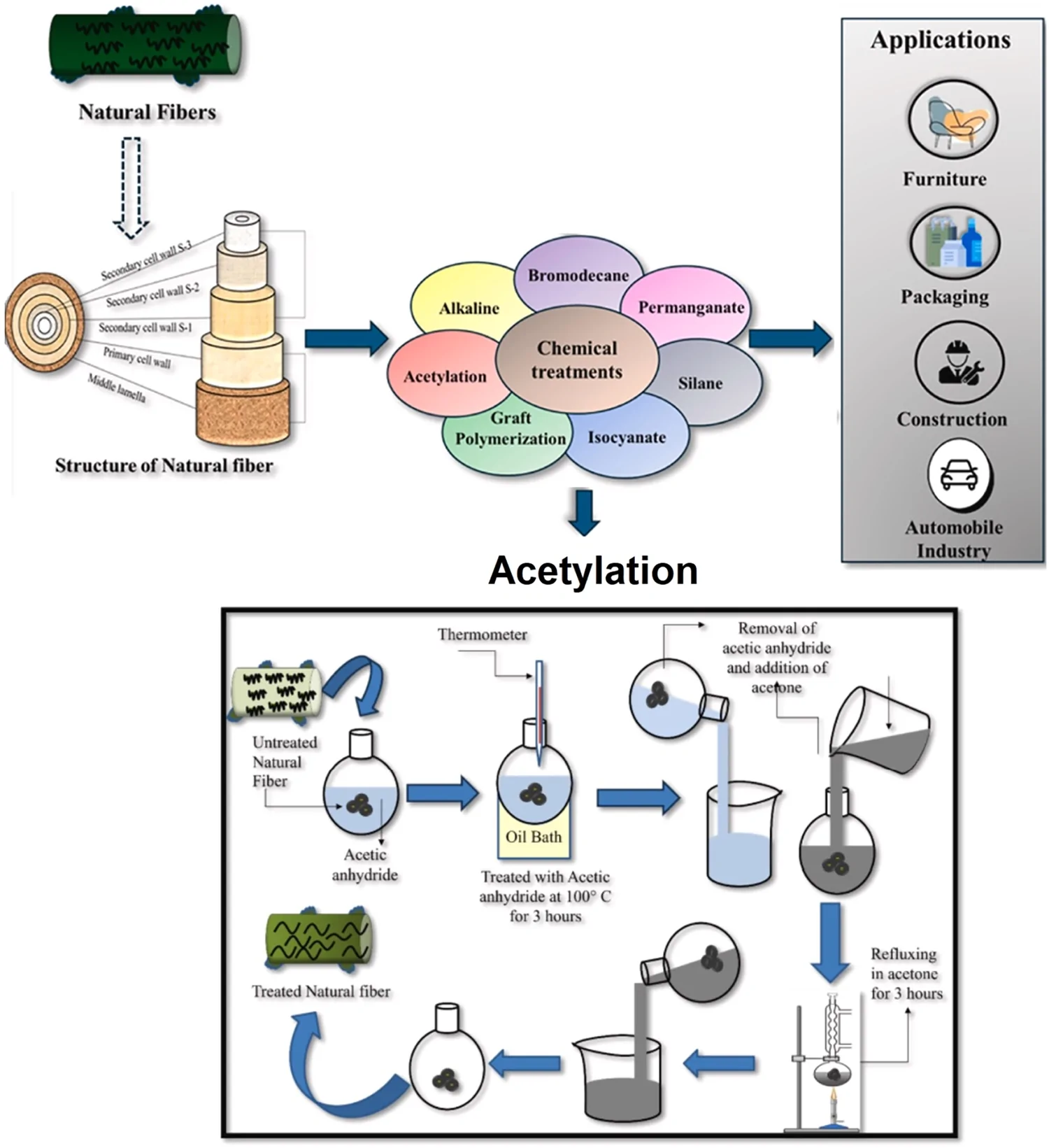
Summarized strategies of the chemical surface modification of the natural fibers for material applications with illustrated acetylation in detail. Reproduced with permission, Copyright 2024, Elsevier.^138^

To answer the initial questions regarding applicability and scalability, natural fibers are best suited for noncritical parts and components used in indoor environments, where they serve as a lightweight alternative to more expensive materials. Surface modification and hybridization (mixing natural and standard fibers in composites) can improve functional properties, but their levels still do not match those of the standard composites. Industrial-scale production is possible; moreover, many natural fibers may be less expensive than commercial materials.

Specific approaches to more sustainable composite reinforcements were described; however, the quantitative impact should also be evaluated, as the measured data reveal the overall efficiency and fundamental character of the proposed strategy. Regarding the reuse/valorization of glass, carbon, or other circular reinforcements, the energy required to recover the solid-phase components is the most crucial factor. The recent circularity study recovering solid-phase reinforcement from wind turbine blades found that the entire industrial process, with an initial input of 100 kg of the original waste material, produces 33 kg of recovered fibers. This strategy requires several industrial steps, such as shredding, particle separation, and pressing. The compound energy and material demands for processing 100 kg of the original material with 33 kg of recovered fibers were 520.69 kWh of electricity, 128.95 m^3^ of natural gas, and 15,137 L of water.^178^ The recovery of energy and carbon fibers from wind turbine blades, comprising unsaturated polyester resin, requires 3.6095 kWh of electricity per kg of input and 2 L of water per kg of input during processing, and produces 20 MJ/kg of pyrolysis oil and 6.7 MJ/kg of gaseous product. In addition to the energetic demands, pyrolysis also involves emissions of CO_2_, CO, NO_*x*_, SO_2_, volatile compounds, and dust.^181^ The environmental impact of natural fiber sources was also quantified in the studies and investigations. The study published by Joshi et al.^179^ uncovered that the nonrenewable energy requirements for glass fiber reinforcement reached 54.7 MJ/kg in total (involving the production of raw materials, transport, melting, industrial production, and other processes), which is significantly excessive compared to the flax fibers (9.55 MJ/kg) and the China reed fiber (3.64 MJ/kg). Another recent study evaluated the carbon, ecological, and water footprint of the different natural fibers compared to the glass fibers, which reached respective values of 2.38 kg CO_2_/kg fiber, 7.60 m^2^·A/kg fiber (where “*A*” stands for Earth’s biologically productive area necessary to produce 1 kg of the analyzed material), and 0.0411 m^3^ H_2_O/kg fiber.^180^ The particular investigated natural fibers are summarized below:

- Cotton Fibers
  - Carbon footprint = 2.95 kg of CO_2_/kg of fiber.
  - Ecological footprint = 31.32 m^2^·A/kg fiber
  - Water footprint = 2.0700 m^3^ H_2_O/kg fiber.
- Jute Fibers
  - Carbon footprint = 0.58 kg of CO_2_/kg of fiber.
  - Ecological footprint = 4.73 m^2^·A/kg fiber
  - Water footprint = 1.5500 m^3^ H_2_O/kg fiber.
- Kenaf Fibers
  - Carbon footprint = 0.55 kg of CO_2_/kg of fiber.
  - Ecological footprint = 4.21 m^2^·A/kg fiber
  - Water footprint = 0.6999 m^3^ H_2_O/kg fiber.^180^

Except for the water footprint, kenaf and jute fibers exhibit considerably lower environmental impacts from a carbon and ecological standpoint. Cotton fibers outperform glass fibers across all three reported parameters, indicating significant processing demands in exchange for the use of biobased, natural composite fabrication.

To efficiently demonstrate differences in material performance and application potential, Ashby plots illustrate Young’s modulus and tensile strength for fiber materials with varying densities, providing a clear comparison of available materials for composites. Generally, glass of basalt fibers exhibits significant Young’s modulus and tensile strengths; however, certain natural fibers match the modulus of the available materials (especially Hemp fiber, see Figure 5), and the particular glass strengths (flax or kenaf fiber), which could lead to prospective composite materials when suitable resin is employed. Moreover, since natural fibers generally exhibit significantly lower densities (1.2–1.5 g/cm3), the lightweight nature of the fabricated heterogeneous materials supports prospective sustainable solutions.

**Figure 5:**
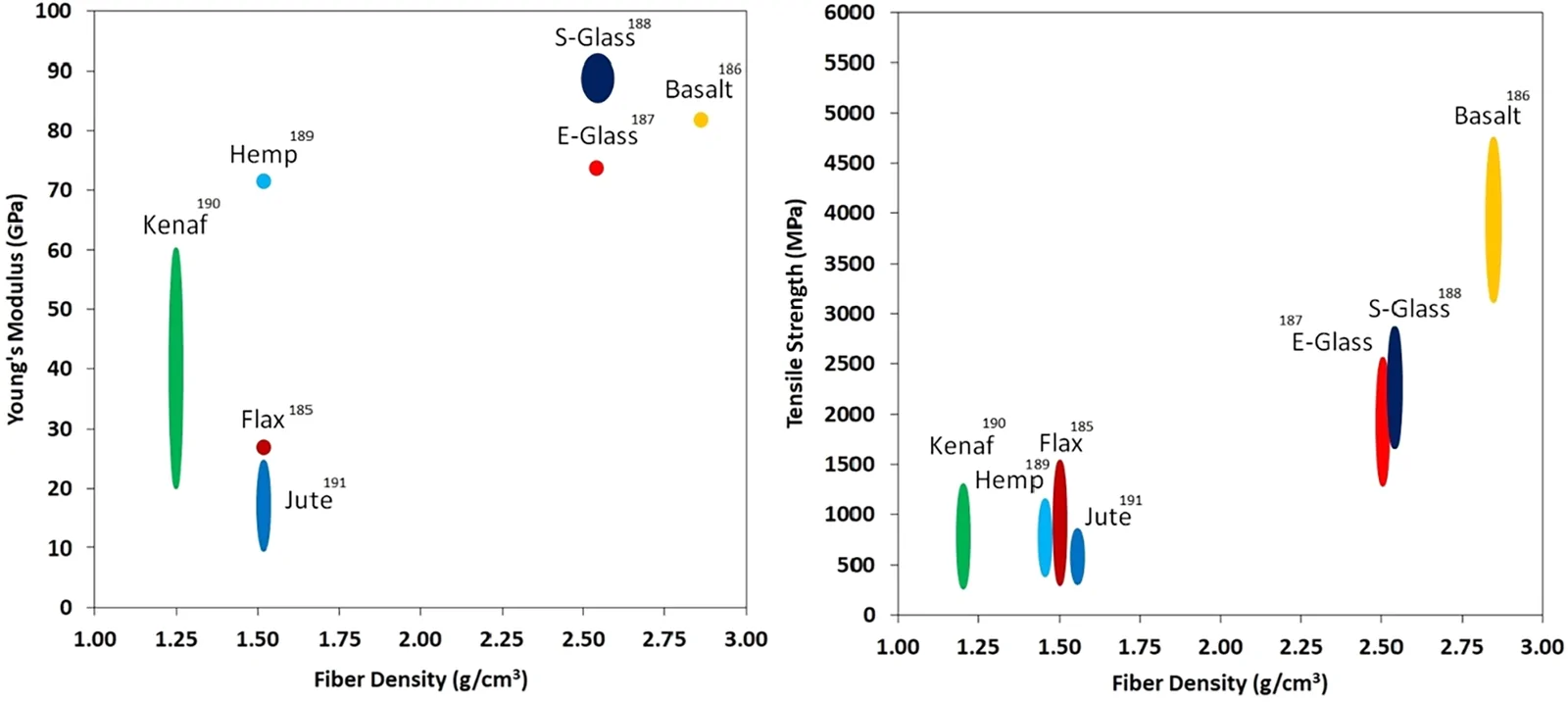
Ashby plot summarizes the relationship between Young’s modulus and tensile strength of the most applied glass and basalt fibers compared to the natural materials, and the densities of the fiber materials.^185–191^

## 3 Sustainable Matrix Approaches

The binding resin accounts for a minor weight fraction in most composite systems, especially in glass fiber-based materials, primarily to achieve cost savings.^140^ Regarding carbon fiber composites, their price is much lower than that of continuous fillers; nevertheless, the binding resin is usually minimized, since most of the superior functional properties are provided by the solid-phase reinforcement.^141^ Nowadays, most of the resins used for composite fabrication come from fossil-based sources, like unsaturated polyesters, vinyl esters, polyurethanes, and epoxy resins.^142^ Multiple ways lead toward increasing the sustainability character of the composite industry. Many polymer-related applications and production wastes may be reused for “down-grade” applications, such as construction or specific automotive composites and laminates. Reused/upcycled thermoplastics, such as PET, PP, or high-density polyethylene (HDPE), are suitable for composite production, enhancing sustainability.^143^ Furthermore, the reactive resin-forming precursors appropriate for composite fabrication can be synthesized from various entering compounds and systems after specific chemical modification.^144^ Any recycled/upcycled material, together with the valorized waste, reduces the accumulation of pollution and boosts the sustainable industry. The particular investigation employing waste thermoplastic inputs was reported in the published literature. PET and recycled PET (rPET) were used in composite production in two recent investigations,^213,214^ which examined the effects on structural anchoring in civil engineering applications and on the incorporation of recycled PET flakes into construction composites. In addition to the PET-containing composites, Rezaei et al.^215^ introduced recycled PET/PC (polycarbonate) glass fiber composites as sustainable alternatives to the currently fabricated virgin thermoplastic composites. Alongside waste valorization and material circularity, novel, highly or fully biobased systems can also lead to environmentally friendly solutions in the composite industry. The structural suggestions and experimental investigations of itaconic acid-based systems exemplify material alternatives produced via a biotechnological approach.^145^ Both valorized and novel biobased resin-forming systems can potentially reduce manufacturing costs or the carbon footprint. This section presents numerous fundamental solutions and critically evaluates their applicability in industry, along with functional comparisons with current composite systems.

The circular application of polymers and systems that no longer fulfill their original roles in composite fabrication not only reduces potentially generated waste but, when incorporated into multicomponent materials, can provide several added values that complicate the original use but are beneficial to composite fabrication. Especially, thermoplastics (such as PET, PE, or PLA) are suitable for recycling and upcycling in the composite industry, as they can be melted and circularly incorporated into multicomponent materials.^146,147^ Moreover, when their functional properties, such as molecular weight, optical appearance, and others, do not reach the standard levels for the original application, most of the composite materials still achieve the required characteristics even with “off-grade” incorporated polymers.^148^ The PET waste is one of the most common streams collected and sorted from the dumpster source and is primarily used for mechanical recycling, producing recycled PET products. Specific PET “off grades” produced by recycling manufacturers, excessively degraded waste, or amorphous PET used regularly in many packaging applications and specific fields cannot be processed by conventional mechanical recycling, and their still useful potential may be used in composite fabrication.^149^ The published literature sources describe numerous eventual utilities of the potentially “off-grade” PET, such as electrospun composite nanofiber mats or sustainable composite acoustic panels.^150,151^ PLA represents a specific role in terms of sustainable or recycled/upcycled composites and multicomponent materials. Polylactic acid (or polylactide) exhibits several challenging properties related to its chemical structure, such as lower thermal stability, a tendency to hydrolyze, and the occurrence of secondary (cold) crystallization at a specific temperature (90–110 °C).^152,153^ Therefore, the original application involving PLA typically cannot use the polyester via a closed-loop strategy an unlimited number of times. The upcycling of PLA waste in the composite industry opens new perspectives for the “off-grade” polymer, with experimentally studied applications such as biodegradable multicomponent films containing PLA or PLA-based cellulose nanocrystal composites.^154,155^ The incorporation of waste or “off-grade” systems and polymers fundamentally increases the sustainability of the process, but the main issue lies in the properties maintenance due to the heterogeneous character of the used waste.^156^ But in terms of specific, suitable application selection, this strategy is undoubtedly scalable and profitable, and composites made from recycled, upcycled, or waste materials attract major attention.

While the valorization of linear thermoplastics represents a straightforward and efficient strategy toward sustainability-increasing manufacturing processes by simply circularly melting and incorporating polymers into multicomponent systems, the chemical engineering suggests that resin/compound modifications before application exemplify a more complex but fundamentally attractive way to produce environmentally friendly materials. Compared to processable polymers, such as PET, PP, or PLA, thermoset resins, which are also often used for composite fabrication (e.g., epoxy and polyurethane), represent cured systems that cannot be melted or conventionally recycled and require specific chemical functionalization before numerous reaction conditions and chemical approaches.^157^ Our research groups recently published studies describing the specific glycolysis of polyurethane waste from the automotive industry using modified castor oil and waste from the cosmetic industry (coconut oil). The performed depolymerization strategies monitored polyurethane decomposition under microwave-assisted, high-temperature conditions.^195,196^ Typically, the hydroxyl value is monitored during glycolysis since free hydroxyls are formed in the liquid raw depolymerized system and released as transesterified polyols.^195,196^ The transesterification kinetic models follow multistep reactions, typically involving the formation of soluble oligomers from the depolymerized particle surface, followed by reactions producing final low-molecular products (both steps reach equilibrium, but heterogeneous depolymerization typically produces oligomers quantitatively).^197^ The acidolysis comprises two separate reactions: urethane structure with acid and urea reaction with acid, producing multiple products (recycled raw material, polyol, CO_2_, and H_2_O), and several aspects affect the acidolysis, such as the p*K*_a_ of the used acid, its molecular weight, or its melting temperature.^198^ Since the standard high-temperature conditions may not be enough for the reprocessing of the thermoset matrices, the microwave-assisted conditions can represent a promising strategy.

In the published literature,^158^ the reprocessing of the epoxy resin used in glass fiber and carbon fiber composites was performed via microwave heating of 160–400 W power in the presence of biobased reactive compounds—hydrogen peroxide (H_2_O_2_) and tartaric acid (C_4_H_6_O_6_). Microwave irradiation provides several advantages over conventional heating systems—specific molecules with high permanent dipole moments tend to increase their molecular motion (leading to temperature increases) very efficiently and in a significantly shorter time than other heating approaches. Moreover, because microwave irradiation affects highly polar systems before reaction with gaseous molecules (unlike conventional oven heating), energy consumption is minimized compared with standard heating.^159,160^ The researchers processing the used epoxy matrix via the described approach achieved the total depolymerization of the resin, and the recycled content was successfully incorporated into the virgin precursors to reduce the primary consumption of the original matrix, along with the waste reduction.^158^ Another closed-loop recycling approach was performed with thiol-functionalized carbon fiber composites cured with epoxy-ended degradable hyper-branched polymer (see Figure 6). The authors achieved highly efficient recyclability (89%) of the used resin via a reactive mixture of phosphoric acid (H_3_PO_4_) or formic acid (HCOOH) dissolved in tetrahydrofuran (THF). The chemical approach using H_3_PO_4_ involved a multistep modification of the depolymerized resin with formaldehyde, phthalic anhydride, and glycidol, producing a reactive precursor capable of circular curing with an amine-hardener and thiol-functionalized carbon fiber to form a recycled composite. The second approach, using HCOOH, involved a much more straightforward recycling process: functionalization with formaldehyde, followed by hot-pressing the recycled precursor with the fibers. In addition, 100% of the originally used carbon fibers were recycled in the proposed strategy.^161^

**Figure 6:**
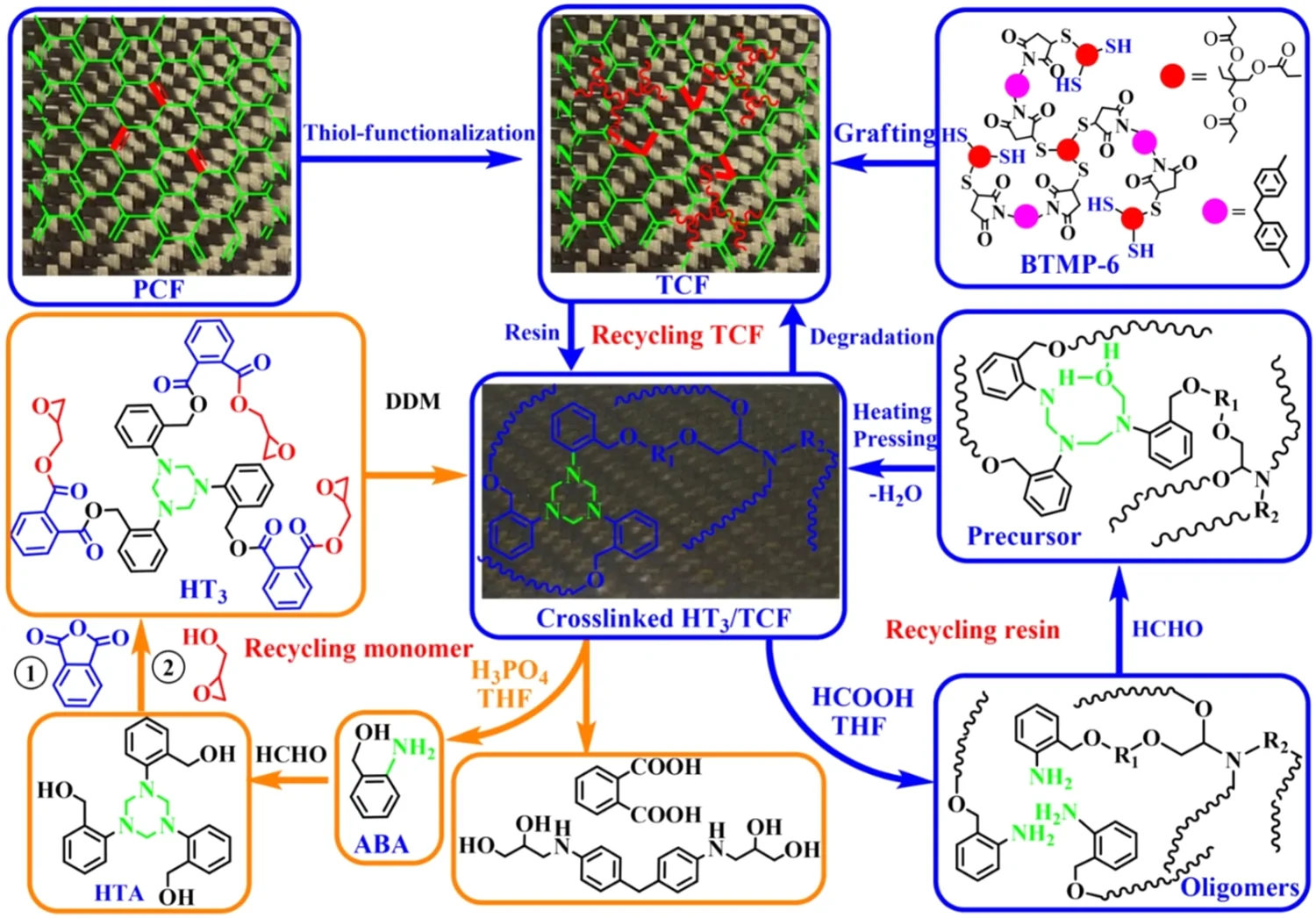
Proposed approach to closed-loop carbon fiber composite recycling involving 100% fiber circularity and 89% recycling of the used epoxy-derived hyper-branched matrix, using two different acidolysis approaches. Reproduced with permission, Copyright 2021, American Chemical Society^161^

Vitrimers represent an attractive and promising chemical system that achieves the material performance of thermosets while retaining reprocessability, enabling their potential circularity. Such engineered materials are also referred to as cross-linked polymers with dynamic covalent bonds that can be reprocessed, typically at elevated temperatures and high pressure.^205^ The original 3D molecular polymer site, exhibiting unique and high-performing material properties, is transformed into a mold, processable state, forming new dynamically forming covalent bonds employing the present functional groups, such as transesterification of the free hydroxyl groups,^206^ nucleophilic substitution employing excess epoxy groups,^207,210,211^ hindered urea bonds,^209^ or disulfide bonds.^208^ Vitrimer systems in composite materials represent high-performing curable matrices with the potential for sustainability through reprocessability. Several experimental investigations focused on employing systems with dynamic covalent bonds in fiber-polymer composites.^210–212^ In the published literature, a polyester system fabricated from epoxidized dicardanol-based succinate cross-linked with hexahydro-4-methylphthalic anhydride was investigated, which contained numerous free hydroxyl groups formed after nucleophilic substitution of the anhydride cross-linker with epoxy functional groups. Such a polar, cross-linked vitrimer was reprocessed after 10 min of hot-pressing at 180 °C.^201^ A different study focused on vitrimers employed in recycled carbon composites also highlighted the reactivity and dynamic covalent character of the epoxy functional groups.^202^ Besides the vitrimers with transesterification mechanism or continual ring opening epoxy nucleophilic substitutions, particular experimental investigations also demonstrated the dynamic covalent character employing disulfide bonds mentioned earlier.^203,204^ Not only do vitrimers exhibit reprocessable structural character, but the formed functional groups in their structure positively affect the interfacial compatibility with the fiber filler; therefore, their performance can reach better results compared to the standard thermoset polymers.

Itaconic acid-based highly and fully renewable reactive precursors for composite matrices have attracted significant attention in current research focused on environmentally friendly systems and materials.^162^ This dicarboxylic acid is produced via a biotechnological approach using various microbial producers, such as *Aspergillus terreus* or *Ustilago maydis*.^163,164^ Due to its production via a sustainable biotechnological process, this compound is currently being increasingly studied, and many products and materials have been reported in the available literature (see Table 2). The polymerizable methylidene group on itaconic acid’s carbon backbone is the main added value of this biobased compound. This reactive functional group enables radical-initiated curing for composite fabrication and 3D printing, as well as for functional coatings and specific heterogeneous hydrogels.^165–167^ Regarding the composite industry, itaconic acid is a perfect substitute for unsaturated polyester systems, widely used as a matrix due to its structural similarity to commonly used maleic acid derivatives (two-functional carboxylic acids capable of radical polymerization).^168^ Since itaconic acid is produced in dicarboxylic form, this chemical structure is slightly complicated from the processing standpoint compared to the usually used petroleum-based carboxylic acids in form on anhydride (maleic anhydride or isophthalic anhydride).^169,170^ The functionalization of itaconic acid requires transformation to itaconic anhydride (which is complicated and industrially nonperspective), or engineering of the Fischer esterification approaches involving condensation reaction with specific supportive reactants (mainly polyols) (see Figure 7).^171,172^

**Figure 7:**
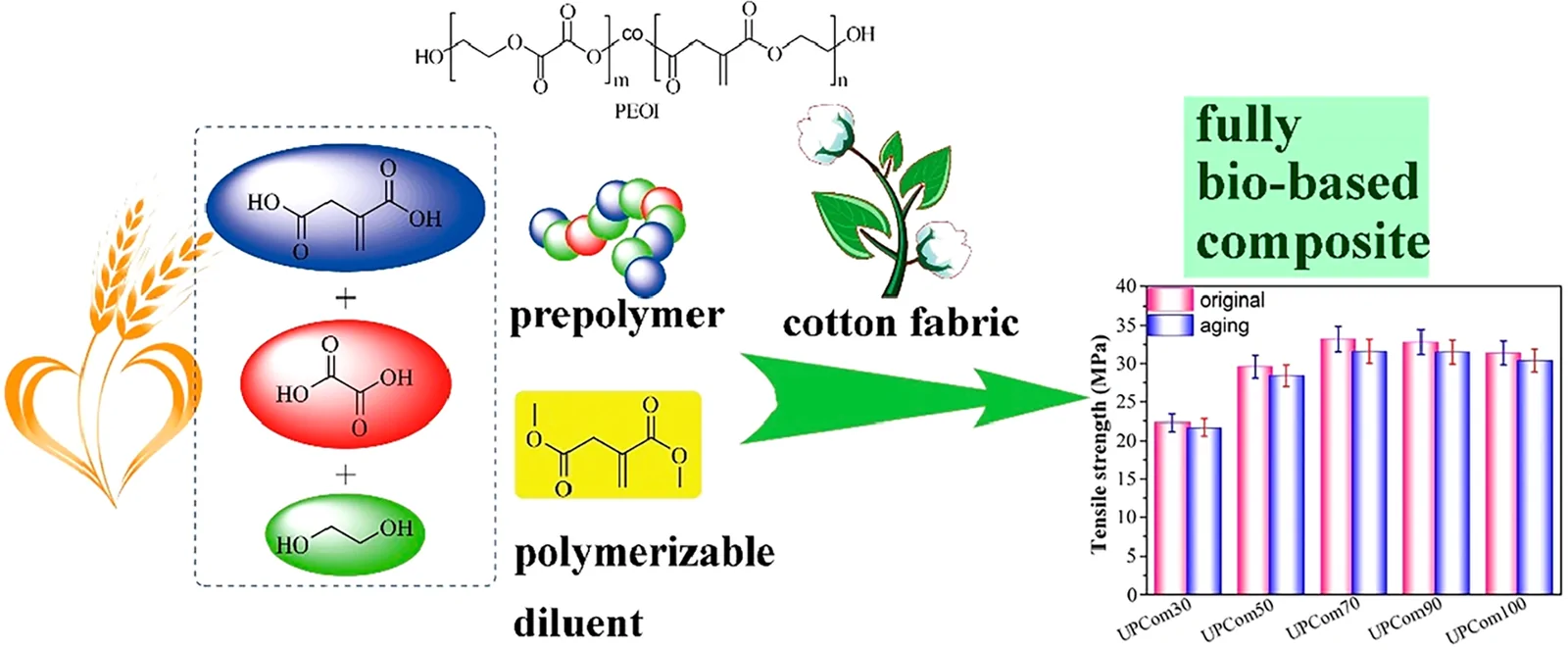
Fully biobased composite, fabricated from the itaconic acid-containing polyester diluted with itaconic acid dimethylester, incorporates cotton fabrics as a natural fiber solid-phase reinforcement. Reproduced with permission, Copyright 2018, American Chemical Society^173^

**Table 2:** Summarized Itaconic Acid-Based Composites and Heterogeneous Materials Representing Highly or Fully Renewable Source-Based Systems

prepared system	involved compounds	application	tensile strength (MPa)	itaconic acid content	reference
fully biobased polyester	itaconic acid, ethylene glycol, oxalic acid	UPEs substitute in construction, transportation, and the marine industries	23–35	15–50%	^173^
itaconic-based sulfur-containing epoxide	itaconic acid, epichlorohydrin	carbon fiber composites, adhesives	35–60	≈40%	^174^
Itaconic-based epoxide	itaconic acid, epichlorohydrin, dicyandiamide, diglycidyl ether bisphenol A	high-performance carbon fiber composites	2600–3050	≈8%	^175^
itaconic acid–based dispersant	itaconic acid, acrylic acid, acrylamide, vinyl sulfonic acid	carbon fiber reinforced composites	35–85	50%	^176^
itaconic-acid-modified polyesters diluted with dibutyl itaconate	itaconic acid, maleic anhydride, poly(butylene succinate)	green composites for potential packaging applications	12.7–29.1	<1%	^177^

Not only do itaconic acid derivatives for curable composite matrices represent sustainable alternatives to the fossil-based unsaturated polyesters, vinyl esters, or epoxy systems, but the application of a reactive dicarboxylic acid obtainable by biotechnological cultivation offers a prospective strategy for independent material manufacture. Due to the wide variety of feedstocks for itaconic acid production, the reliance on imported petroleum compounds can be reduced, ensuring greater control over the production of engineered resins. In addition to this advantage, the overall lower hazardous character of IA and its derivatives also supports the potential replacement of primarily unsustainable precursors in the composite industry. Dangerous volatile compounds are a significant issue associated with the production of common polymer-fiber composites. The low-molecular-weight itaconates and polyester macromolecular systems represent reasonable alternatives to the current polymerizable precursors.

Since the purpose of this perspective is to provide a reasonable insight into the most promising approaches and strategies for processes that are applicable, economically rational, and efficiently scalable, several points regarding environmentally friendly possibilities involving composite matrices need to be summarized. In this section, we initially discussed options involving circular application of waste and “off-grade” thermoplastics for composite manufacture (PET, PLA, PE, and others). This approach exemplifies the most promising approach for the future up-scaled industry sector. The selected waste and nonperforming conventional fossil- or biobased thermoplastics represent, to varying degrees, an available entering stream for the composite matrix systems. The main adverse property of waste-source systems is their varying structural quality. Several properties, such as molecular weight, dispersity, and oxidation stage, must be monitored to achieve consistent quality in the produced composites. Then, chemically modified resins and thermoset matrices were discussed, which mark the potential for closed-loop circularity in fiber composites. Many fabric-containing materials are manufactured from thermoset materials, which are usually highly performing but difficult to valorize. Particular strategies, involving microwave-assisted depolymerization or acidolysis, were introduced; however, the multistep nature of these processes increases their complexity and scalability. Although closed-loop circularity is one of the most attractive approaches to achieving highly sustainable manufacturing, all of the steps outlined must be considered. Eventually, the partially or fully biobased matrices derived from itaconic acid were summarized throughout this perspective to provide a novel and innovative strategy toward sustainable composites. Numerous polyester and epoxy reactive systems have been investigated in the published literature as alternatives to petroleum-based resins. Although these materials use renewable, sustainable starting materials and, in many cases, also exhibit exceptional functional properties, their overall cost would be a major issue for potential industrial upscaling. Also, itaconic acid is mainly used in a dicarboxylic form, which requires high-temperature condensations (for polyesters) or nucleophilic substitutions via epichlorohydrin (for epoxides). Both processes are either high-energy consumers or pose greater environmental and health hazards. Overall, the biggest potential lies in incorporating waste materials, preferably selected thermoplastics, rather than chemically modified resins or biobased itaconates.

Economic factors significantly influence the potential utility of biobased matrices. The recently reported cost of itaconic acid is stabilized at 1.5–2.0 USD/kg.^182^ As described earlier, several biobased curable systems demand several additional compounds and reactants to form a competitive polymerizable matrix. Ethylene glycol, as a major contender for polyester itaconic systems, costs around 0.5–0.6 USD/kg, which must be incorporated into the eventual economic balance.^183^ On the other hand, the costs and requirements for chemically recycled or upcycled resins compose the price for waste input and also the assisting chemicals used for the fabrication. For example, PET is used for mechanical recycling and as a recyclable polymer for composite fabrication.^147,148^ The cost of PET waste fluctuates significantly due to the industrial material market and demand. The reported PET scrap price in the USA in Q1 2026 reached 1.27 USD/LB (2.8 USD/kg).^184^ Regarding cost efficiency, chemical recycling and upcycling require energy consumption, similar to the synthesis of virgin biobased polyester resin; however, the main parameter is the market waste cost. A much more efficient approach is the direct reprocessing of waste thermoplastics into thermoplastic composites; however, this strategy is highly dependent on the quality and purity of the input material and requires specialized apparatus for processing hot melts rather than liquid-curable systems.

Considering sustainability and the most promising future impacts of recycled or biobased curable resins on the materials industry, these points represent rational, promising strategies for enhancing the composite industry. Given the nature of chemical recycling and upcycling, these approaches should be applied primarily to recyclable inputs that cannot be further reprocessed by mechanical recycling or direct remodeling. Several depolymerization and modification concepts can valorize highly impure heterogeneous mixtures that would otherwise be incinerated or landfilled. Material valorization should primarily focus on underutilized inputs and avoid competing with more efficient and effective approaches to ensure sustainable processing. The second idea targets the biobased reactive precursors used for the composite industry. Since the costs and requirements of fully biobased systems entail many processing and chemical aspects, the engineering of fully renewable materials is preferable to incorporating minor or partial “green solutions”. Due to the complexity of the process and the reliance on fossil-based compounds, entirely natural-based solutions offer attractive, highly sustainable materials, reduce society’s dependency on imported sources, and provide unique systems with unobserved properties. The biobased reactive matrices should be derived primarily from renewable sources rather than labeling themselves “green materials” with negligible bioderived content, only to contain fossil-based, dependency-forming compounds.

## 4 Conclusion and Future Outlook

This work summarizes numerous potentially promising or fundamentally innovative strategies for more sustainable and environmentally friendly fiber-based polymer composites. Nowadays, glass fibers, carbon fibers, aramid, polyamide, and other commercially used materials are the main reinforcements in most functional composites. The fibers themselves are usually stable, high-performance materials that retain their properties even after manufacture and long-term use. Therefore, their circular reuse exhibits a prospective strategy toward sustainability, not only because of their sufficient functional properties but also because of the waste reduction associated with circular valorization. We described the incorporation of reprocessed fibers, the formation of powders, and the valorization of entire composites into enhancing and functional reinforcements and fillers that can be incorporated into new composites. The application of natural fibers is another approach toward environmentally friendly multicomponent materials, which exhibit both positive and adverse characteristics. Natural fibers are generally less expensive than high-performance S-glass or carbon fiber reinforcements and usually have a significantly lower overall density. Therefore, natural fiber-based composites may successfully serve in interior, decorative, and nonfunctional applications that require cost-efficient fabrication and are moving toward sustainability. On the other hand, natural fibers will never match the performance properties of glass or carbon fibers and fabrics; therefore, their functional character is limited. Also, since lignocellulose fibers are highly hydrophilic, their surfaces must be modified to improve matrix-reinforcement compatibility and long-term stability. Because of their lower density, natural fibers represent a prospective alternative in the automotive or aerospace industries, offering the potential for weight reduction. In contrast, recycled glass and carbon fibers hold enormous potential in the construction and energy sectors (glass fibers) due to their cost-effectiveness, and recovered carbon fibers and particles find applications in high-performance, sustainable composites.

The matrices used in the composite industry are usually smaller but equally important components of the resulting composite materials. Numerous strategies enhance the sustainability of resins for composites, such as incorporating waste and “off-grade” thermoplastics, such as PET, PLA, or PE. The valorization of sorted conventional plastic scraps exemplifies the most promising approach due to the readily available source stream, low cost, and convenient processability. The heterogeneous nature of the incorporated polymer waste is one of the most significant issues associated with this approach. The processability of thermoset resins is significantly more complex than that of thermoplastics. Highly complex chemical strategies and processing conditions must be applied to obtain a useful system that can be incorporated into the new composites. On the other hand, such procedures deal with practically permanently stable polymer networks that form a cross-linked resin. The approaches, such as microwave-assisted depolymerization or acidolysis, lead to reprocessable systems. Although such processes involve multistep modifications, these approaches result in closed-loop circularity, which is fundamentally significant. The application of partially or fully biobased resins derived from itaconic acid offers an innovative approach to sustainable composites, but manufacturing costs may be the Achilles’ heel of this process. The upcycling and recycling of spoiled and waste polymers and systems find their prospective utility in heterogeneous waste mixtures with numerous impurities—due to the selectivity of the depolymerization reaction, the heterogeneous character usually does not negatively affect the process. The produced matrices are primarily used in industrial-scale production. On the other hand, partially and fully biobased composites, often exhibiting lower health and environmental hazards, hold enormous potential for in-contact applications and commercial products.

The polymer-fiber composites represent a large and complex group of material combinations, numerous reinforcement-matrix variations, and several additives and enhancers that affect the final material properties, which were not discussed in this Perspective. This work focuses primarily on the two major components in composite materials but lacks discussion of solid-phase additives, flow modifiers, separators, polarity enhancers, reactive diluents, and many other important components. The perspective offers several strategies to enhance sustainability in the composite industry, but the resulting heterogeneous material must be functional across all incorporated components.

Considering the full integration of sustainable approaches and strategies discussed throughout this perspective, many aspects and limitations related to material changes and the use of innovative compounds must be taken into account. We summarize essential points to consider for a successful transformation of the current composite industry toward a more sustainable future in heterogeneous materials fabrication.

- **Limited material characterization due to the absence of standard renewable systems**—as described throughout this work, several natural fibers (primarily) and biobased matrices (minor) often vary in measured material characteristics due to different quality of the entering source (reinforcement) or nonconstant level of biobased resin polymerization (affected by biobased monomer sources, water content, or contaminants). Compared to the silanized E-glass/vinyl ester systems, which are described meticulously, the suggestion of standard composite systems comprising renewable components would set the right direction for the development of invented and engineered materials.
- **Development of functional metrics specific to renewable composites**—sustainable systems exhibiting a positive effect on carbon or environmental footprint would benefit from a set of normalized metrics comparing them, selecting the best-performing candidates ideal for industrial scaling. Besides the discussed metrics, such as carbon, environmental, and water footprints, parameters such as environmental impact per functional unit or per functional duration would put sustainable materials into perspective with other systems.
- **Life cycle assessment (LCA) combined with performance indicators**—to combine LCA with the eventual performance of sustainable composites could represent a comparable indicator for currently used materials and the developed systems.
- **Specific case studies**—specific composites comprising biobased and renewable sources or recycled content could be applied in less performance-demanding applications (interior/decorative parts, covers, and supportive devices). Special case studies focused on selected applications could uncover the best substituting potential of renewable composites in the material industry.

Each sustainable approach yields a different composite material that possesses either a highly environmentally significant aspect or a unique role for a particular product or manufacturing process. The combination of the function, economy, and industrial applicability is key to selecting a prospective strategy that is optimal for the specific application.
